# Radiographic results on acetabular cup placement with the SuperPath technique: a retrospective study of 756 cases

**DOI:** 10.1186/s12891-022-05065-7

**Published:** 2022-01-31

**Authors:** Agostino Di Maro, Santo Creaco, Mattia Albini, Mahfuz Latiff, Marco Merlo

**Affiliations:** 1 Department of Orthopaedics and Trauma Surgery of Ospedale di Circolo Busto Arsizio, ASST Valle Olona, Via Arnaldo da Brescia,1, Varese, Italy; 2 Department of Orthopaedics and Trauma Surgery of Ospedale di Circolo Fondazione Macchi Varese, ASST Sette Laghi, Viale Borri 57, Varese, Italy

## Abstract

**Background:**

The Supercapsular percutaneously assisted total hip (SuperPath) technique is a relatively new minimally invasive approach for total hip arthroplasty (THA). Good clinical outcomes related to its use are reported in the literature. Nonetheless, there are still uncertainties about its validity in terms of radiographic outcomes.

Main purpose of the study is to evaluate the effectiveness of the SuperPath in acetabular cup positioning through radiographic evaluation of acetabular inclination angle (IA) and acetabular anteversion (AA) angle within the safe zone described by Lewinnek. The leg length discrepancy (LLD), femoral offset (FO), and acetabular offset (AO) were also measured to ascertain the radiographic effectiveness of SuperPath in the acetabular cup placement.

**Methods:**

Between January 2016 and December 2019, all SuperPath cases eligible for the study were included. They were operated by three orthopaedic surgeons with long-standing experience in THA via conventional posterolateral approach and who have performed SuperPath training fellowship. The Mann-Whitney U test was used for statistical assessments (*p*-value < 0.05). Means ± standard deviation (SD) of the radiographic IA and AA were calculated for each year.

**Results:**

A retrospective analysis of 756 THAs was performed. The average percentage of IA within the Lewinnek’s safe zone was from 80 to 85%, while the average percentage of AA was from 76 to 79%. Both IA and AA showed no statistically significant difference between two consecutive years. Good results, in the ranges of normal values, were also obtained for LLD, FO and AO, with homogeneous outcomes between 1 year and the following one.

**Conclusion:**

It is possible to achieve good radiographic values of acetabular cup orientation through the SuperPath within the Lewinnek’s safe zone. These results are similar to those reported in the literature by authors using SuperPath. Low rate (0,3%) of hip dislocations were reported. Therefore, the SuperPath technique represents a good alternative THA approach. Nevertheless, there is not a statistically significant improvement in these radiographic parameters over a four-year time.

**Level of evidence:**

Level IV, retrospective study.

## Introduction

The correct placement of the acetabular cup in total hip arthroplasty (THA) surgery is associated with better clinical outcomes and a lower complication rate [1–8]. Several surgical approaches for THA were developed with the goal of gaining better exposure for placing the acetabular component correctly and safely in all orientations [9, 10]. In addition, a wide variety of surgical minimally invasive (MIS) approaches have gained popularity among orthopaedic surgeons with the aim of obtaining simultaneous satisfactory clinical-radiographic results and greater muscles sparing during the surgical approach [11–14]. Nevertheless, some MIS techniques were associated with higher rates of component malposition, ascribing to a reduced visualization of the acetabulum compared with standard procedures [15–20]. Among the several MIS, the Supercapsular percutaneously assisted total hip (SuperPath) (MicroPort Orthopedics Inc., Arlington, TN, USA) is a minimally invasive technique that utilizes a muscle-sparing surgical approach between the piriformis and gluteus minimus muscles, preserving the insertion of the extrarotator muscles, the posterior capsule, and avoiding the surgical dislocation of the femoral head [21]. Several benefits were attributed to its use such as tissues preservation, early postoperative recovery from the pain, short incision length, short hospitalization, early recovery of daily activities, less perioperative blood loss and a lower transfusion [22–26]. There is not yet a large radiographic case series in the literature regarding acetabular cup placement in THAs via the SuperPath technique.

The purpose of our study is to evaluate the effectiveness of this technique in properly positioning the acetabular cup, through evaluation of the radiographic parameters of the acetabular inclination angle (IA) and the acetabular anteversion angle (AA) of 756 THAs operated in 4 years. The hypothesis of our study is that most cases analyzed have values within the “safe zone” according to Lewinnek, with a good percentage comparable to those obtained using conventional approaches [27]. Lewinnek et al. suggested a “safe zone” of AI of 40° ± 10° and AA of 15° ± 10° to minimize the risk of dislocation. We assessed whether there was a statistically significant difference in this regard between two consecutive years. We also retrospectively examined hip dislocation cases operated over 4 years and with mean follow-up of 30,3 months. We also wanted to investigate if there could be a correlation between the obtained values of IA, AA, the number of cases within Lewinnek’s safe zone and the treated disease (osteoarthritis, femoral head necrosis, fractures, hip dysplasia). In addition, for each year we reported the means of other radiographic parameters such as the leg length discrepancy (LLD), femoral offset (FO), and acetabular offset (AO), with the aim of evaluating the further radiographic effectiveness of the SuperPath technique. Finally, looking at the latest studies reported in the literature describing a comparison between SuperPath and conventional approaches, we wanted to assess where our results of IA and AA could fit in.

## Materials and methods

Between January 2016 and December 2019 at Ospedale di Circolo di Busto Arsizio, ASST Valle Olona (Italy), the SuperPath technique was used for 800 THAs. Exclusion criteria were follow-up less than 1 year, and inadequate radiographic projections for the evaluated parameters [5, 28–30]. Patients included in the study were operated by 3 orthopaedic surgeons who had several years of experience in hip arthroplasty with the conventional postero-lateral approach [31]. They had also performed a fellowship program on the SuperPath technique prior to this study. For each year, we calculated the total number of operated hips, the mean of the IA, AA, LLD, FO, AO values ± SD, and the number of radiographic cases (with associated percentage) that fell within Lewinnek’s “safe zone”. We retrospectively analyzed all radiographic images included in the study for cases of hip dislocations. In addition, it was assessed whether there was a statistically significant improvement or worsening in obtaining these values between 1 year and the following year.

### Radiographic evaluation

**Fig. 1:**
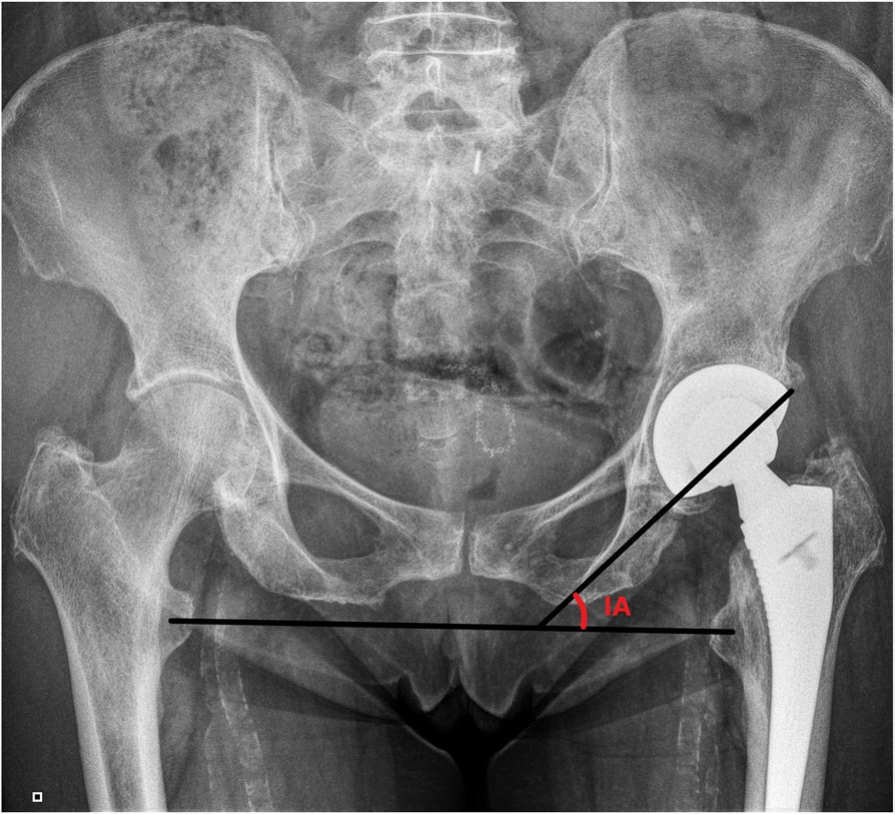
IA measurement. A case of THA with posteroanterior X-ray image shows the measurement of IA. See text for the description of this angle

**Fig. 2:**
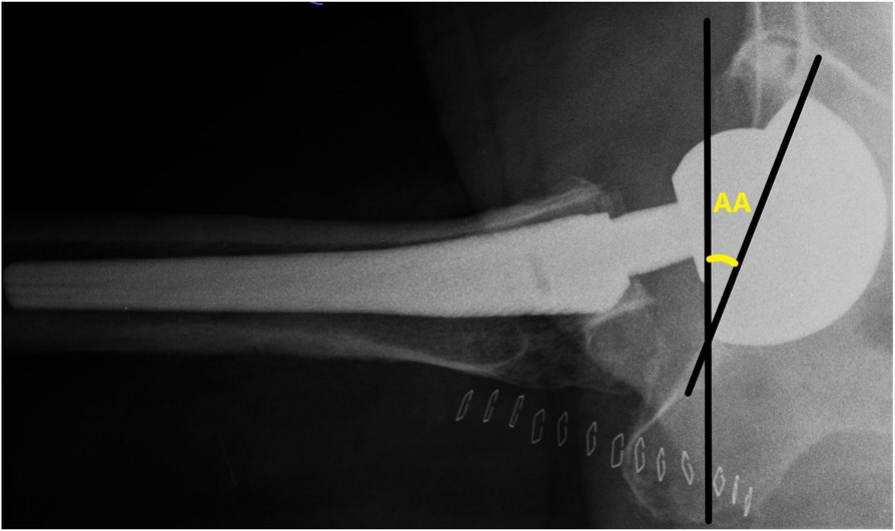
AA measurement. A case of THA with cross-table lateral radiograph for the measurement of AA. See text for the description of this angle

**Fig. 3:**
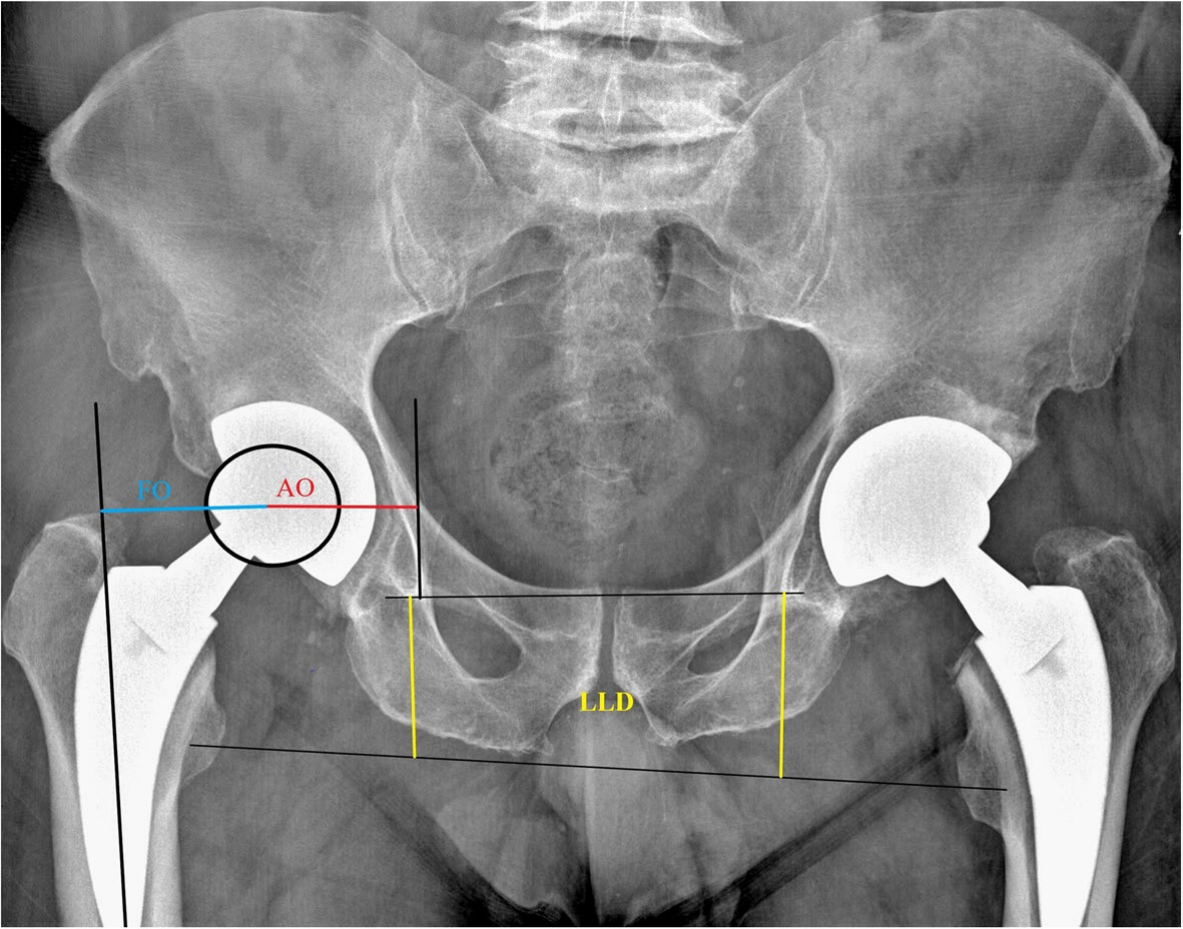
LLD, FO, and AO measurements. An example case of THA showing measurements of LLD (yellow lines), FO (blue line), and AO (red line) in posteroanterior X-ray. See text for the description of these measurements

The primary radiographic parameters considered in our study were the IA and the AA. IA was calculated on a standard posteroanterior radiograph, using the horizontal inter-teardrop line and the oblique line passed through the major axis of the acetabular cup (Fig. 1) [32–34]. AA was measured using a cross-table lateral radiograph, with the angle obtained between the axis of the coronal plane and the axis of the acetabular cup (Fig. 2) [28, 29]. Secondary radiographic parameters evaluated on posteroanterior X-ray were LLD, FO and AO (Fig. 3). LLD was measured as difference of the perpendicular lines drawn from the pelvic reference line (the line transversely connecting the inferior borders of the acetabular tear drops) to the femoral reference line (represented by the line connecting the lesser trochanters) [35, 36]. FO was measured as the perpendicular distance from the center of rotation of the femoral head to the long axis of the femoral canal [37]. AO was measured as the distance from the center of rotation of the femoral head to the perpendicular line passing through the medial edge of the ipsilateral terardrop [38]. The mean (in degrees) of the values obtained ± standard deviation (SD) was then calculated. Patients were evaluated with posteroanterior and lateral hip x-rays at 1 month, 3 months, 6 months, and then annually after surgery. The radiographic parameters were measured by two authors (M.A. and M.L.) on the postoperative images, evaluating the radiographic images extracted from the General Electric Centricity Picture Archiving and Communication System (PACS).

### Statistical analysis

The Mann-Whitney U test was used for the statistical analysis, with a *p*-value < 0,05 considered statistically significant and reported as two-tailed. Values are expressed as mean ± SD.

### Surgical technique

The patient was positioned in a lateral decubitus position, held by appropriate supports, with the hip flexed at 45° and intrarotated at about 10–15°. After setting up the surgical aseptic field, a direct surgical incision was made at the level of the great trochanter in line with the femoral axis. The fibers of the gluteus maximus muscle were spread with a Zelpi retractor. The muscle interval between the gluteus medius and piriformis was reached, capsulotomy was performed in line with the femoral neck, and surgical exposure at that level was maintained using a Romanelli retractor. The femoral canal was reamed using the trochanteric fossa as a reference point (with the starting point of the reaming anterior to the trochanteric fossa) and using a canal feeler to optimally ensure the location of the femoral canal itself. Progressive broaches were used, with the last of them left inside the femur and checked with intraoperative X-rays to assess the exact position. With the last broach into the femoral canal, used as a guide, the femoral neck was cut and the head was extracted with a Schanz pin. A Zelpi retractor was placed subperiosteally and a Romanelli retractor was placed intraarticularly for the acetabulum visualization, an Alignment Handle and a Portal Placement Guide were used so that the top of the guide was perpendicular to the patient’s torso, and the shaft of the guide was tilted approximately 10–15° from vertical to assess pelvic tilt on the operating table. At the point of the intersection of the Blunt Trocar with the leg, a 1 cm mini-incision was made. The blunt trocar was introduced through the mini-incision, posterior to the proximal third of the femur, and the cannula for the acetabular reamer drive shaft was introduced, just posterior to the trochanter as planned for the acetabular placement. The acetabulum was reamed with progressive reamers and then the trial acetabular component was inserted. Trial heads and necks were used according to the preoperative planning, to obtain adequate length of the lower limbs between them and stability of the prosthetic components. After choosing the size of the prosthetic components, the trials were removed, and the definitive components were implanted, ultimately considering visual anatomical landmarks such as the native acetabular version and the transverse acetabular ligament. Final hip maneuvers were performed to assess the final stability of the prosthetic implant. After lavage with saline solution, suturing was performed by anatomical layers [21, 39].

## Results

Out of 800 hips operated, 44 were excluded because 30 had not performed the minimum 1-year follow-up, 2 had died, and 12 had not made adequate radiographic images in order to perform the correct radiographic measurements. From January 2016 to December 2019, a total of 756 hips (719 patients) were enrolled in this study, of whom 350 were male and 369 were female. Thirty-seven patients were operated on both hips, of whom 12 were operated in a single surgical step (single anesthetic), while the remaining 25 patients were operated in two different surgical steps (two separate anesthetic). The mean age at the time of surgery was 71 years (range, 26–104 years). The age of patients operated on both hips was calculated twice at the time of the surgery. The diseases from which the patients were affected were as follows: primary hip osteoarthritis, secondary osteoarthritis, femoral head avascular necrosis, developmental hip dysplasia, and femoral neck fracture. Table 1 summarizes the main patients’ demographics.

**Table 1:** Patients’ demographics

**Demographic charateristics of the patients**	
**Total number of patients**	719
Male	350
Female	369
**Mean age at time of surgery (range)**	71 (26–104)
**Total hips**	756
Primary Hip Osteoarthtritis	454
Secondary Hip Osteoarthtritis	100
Femoral Head Avascular Necrosis	70
Developmental Dysplasia of the Hip	29
Femoral Neck Fracture	103
**Year**	**Number of hips**
2016	164
2017	203
2018	188
2019	201

In 2016 the average IA was 39° ± 8,3; in 2017 the average was 42,9° ± 8,3; in 2018 it was 45° ± 8; in 2019 the IA was 43,1° ± 7,3. In addition, in the year 2016, 80% of the hips operated on were within Lewinnek’s “safe zone” limits; in 2017 that percentage was 82%; in 2018 it was 81%; in 2019 it was 85%. It was found that there was not a statistically significant difference in IA values between 1 year and the next one. The average AA values obtained were 20,2° ± 6,7 in 2016; average values of 15,3° ± 7,1 in 2017; average values of 17,9° ± 8 in 2018; and average values of 16,1° ± 5,8 in 2019. The average annual percentage of hips in the Lewinnek safe zone were: 76% in 2016; 75% in 2017; 79% in 2018; and 78% in 2019. As with IA, we found no statistically significant differences between two consecutive years for AA. Neither a statistically significant difference was found between the first and last years for both IA and AA (*p*-value 2016 vs 2019 for IA: 0,7; *p*-value 2016 vs 2019 for AA: 0,6). Tables 2 and 3 summarize the results obtained.

**Table 2:** Results of IA

Years	Inclination angle mean (°) ± SD (range)	Number (%) of hips within the Lewinnek safe zone	***p***-value	
2016	39 ± 8,3 (18,2 - 63,6)	132 (80)	2016 vs 2017	0,5
2017	42,9 ± 8,3 (21,7 - 63,8)	166 (82)		
2018	45 ± 8 (22–68,8)	152 (81)	2017 vs 2018	0,9
2019	43,1 ± 7,3 (26,3 - 62,3)	170 (85)		
2016 to 2019	42,7 ± 8,2 (18,2 - 68,8)	620 (82)	2018 vs 2019	0,8

**Table 3:** Results of AA

Years	Anteversion angle mean (°) ± SD (range)	Number (%) of hips within the Lewinnek safe zone	***p***-value	
2016	20,2 ± 6,7 (10,1–40)	125 (76)	2016 vs 2017	0,4
2017	15,3 ± 7,1 (5,4 - 51,2)	152 (75)		
2018	17,9 ± 8 (9,8 - 59,3)	149 (79)	2017 vs 2018	0,7
2019	16,1 ± 5,8 (10,5 - 44,6)	157 (78)		
2016 to 2019	17,6 ± 3,2 (5,4 - 59,3)	583 (77)	2018 vs 2019	0,6

Regarding the correlation of results between the treated disease and values of IA, AA and cases within the Lewinnek safe zone, we found overlapping values, i.e. the disease under examination did not represent a factor influencing the radiographic results. Only a slightly better outcome was found in hips affected by primary hip osteoarthritis (84% of cases of IA and 78% of cases of AA in the safe zone). Table 4 summarizes these results.

**Table 4:** Correlation between the hip disease and radiographic results

Hip disease	IA mean (°) ± SD (range)	AA mean (°) ± SD (range)	Number (%) of hips within the Lewinnek safe zone	
			IA	AA
Primary hip Osteoarthritis	40,6 ± 5,6 (33,5 - 58,3)	16 ± 7,9 (14,9 - 31,1)	381 (84)	354 (78)
Secondary hip Osteoarthritis	43,9 ± 6,1 (30,4 - 59,6)	15,4 ± 4,8 (8,9 - 41,3)	82 (82)	76 (76)
Femoral head avascular necrosis	39,5 ± 3,7 (20,5 - 65,1)	14,8 ± 2,5 (5,4 - 40,8)	56 (80)	54 (77)
Developmental hip dysplasia	38,1 ± 7,7 (18,2 - 68,8)	13,2 ± 4,2 (9,8 - 59,3)	23 (79)	22 (76)
Femoral neck fracture	40,1 ± 8,9 (22,2 - 63,6)	16,5 ± 7,1 (9,4 - 46,8)	82 (80)	76 (74)

As mentioned above, we measured the mean ± SD of LLD, FO, AO for each year (including ranges). Homogeneous values were observed between one year and the next, with no statistically significant differences. Table 5 summarizes the values obtained.

**Table 5:** Results of LLD, FO and AO

Years	LLD mean (mm) ± SD (range)	FO mean (mm) ± SD (range)	AO mean (mm) ± SD (range)	***p***-value			
				Years	LLD	FO	AO
2016	2,5 ± 4,1 (−11; 11)	44 ± 6,5 (25–60)	33,7 ± 4,1 (26–44)	2016 vs 2017	0,5	0,2	0,7
2017	3,9 ± 3,4 (−8; 8)	45,8 ± 5,5 (38–63)	33,9 ± 5,2 (24–44)				
2018	2,2 ± 5,2 (−9; 10)	45,9 ± 7,6 (31–70)	33,7 ± 4,9 (26–47)	2017 vs 2018	0,3	0,2	0,8
2019	2,6 ± 3,6 (− 8; 12)	46,1 ± 7,2 (34–65)	34,1 ± 4,8 (27–46)				
2016 to 2019	2,7 ± 4,8 (− 11; 12)	45, 7 ± 6,3 (25–70)	33,8 ± 4,8 (24–47)	2018 vs 2019	0,4	0,3	0,8

In the x-rays analysis, in a mean follow-up of 30,3 months, we only encountered two cases of hip dislocation operated in 2016, approximately 6 and 8 months after surgery.

## Discussions

This series claims that the minimally invasive SuperPath technique allows good radiographic results of acetabular cup positioning, according to Lewinnek’s safe zone. Dislocation rates are low. No significant radiographic differences in acetabular cup positioning were found in two consecutive years. The correct positioning of the acetabular cup was also validated by obtaining values in the normal range of LLD, FO and AO. Malposition of the acetabular cup is associated with complications such as impingement, recurrent dislocation, increased ischial osteolysis and wear of prosthetic components [40–42]. Few studies have evaluated acetabular cup placement depending on the surgical approach. Debi et al. reported IA and AA values using the anterolateral and direct anterior approaches. With the first mentioned approach the IA and AA were 36,5° and 11,3°, respectively. While with the direct anterior approach an IA of 38,3° and AA of 15° were obtained [33]. Moskal et al. reported IA values of 43,57° and AA values of 20,24° using the conventional approach, with a dislocation rate of 2,49% [43]. Soderquist et al. reported IA values of 43,5° ± 7, and AA values of 10° ± 3,1. The dislocation rate was 0,31%, one of which was reported as posterior (nonsurgical reduction), while another dislocation, following a fall, was treated surgically [44]. From a study evaluating dislocation rates in THAs from the Swedish hip arthroplasty register, an increased risk of dislocations was found using minimally invasive and posterior approaches compared with the lateral approach. The interpretation of these results, however, appears to be questionable by assessing other reported studies [40]. MIS techniques arose with the simultaneous effort to reduce periarticular tissue damage and achieve good clinical-radiographic results, the latter at least overlapping with those obtained with conventional surgical approaches [45–47]. In this regard, it would also be appropriate to clarify the correct definition of MIS, since the surgical approaches are manifold and different from each other. This is not the aim of our study, but it would at least provide a better understanding of SuperPath in that framework. Controversies exist over the precise definition of a minimally invasive approach, as no clear definition is reported [22, 41, 48]. The SuperPath technique fits into the group of minimally invasive muscle-sparing techniques, since it preserves the cutting of the extrarotator muscles, hip joint capsule, and avoids surgical dislocation of the femoral head [46]. Several studies report benefits related to this technique [22, 23, 47, 49–51]. Cost-saving benefits associated with the use of the SuperPath were also shown by Gofton et al., as a 28% reduction in in-hospital costs was reported compared with the standard lateral surgical approach [49]. Della Torre et al. reported good radiographic results within Lewinnek’s safe zone, as a mean IA of 40,13° ± 6,30 was obtained from 66 postoperative radiographs [21]. Kay et al. reported zero cases of dislocation in a 2-year follow-up. In addition, a low blood transfusion rate (3,7% of patients), a low hospital stay in 75,7% of cases (2,3 ± 1,0 days), and good radiographic findings of IA (43,6° ± 5,2) and AA (20,9° ± 6,2) were obtained [52]. Ramadanov et al. showed, in a systematic review and network meta-analysis of randomized controlled trials, that the SuperPath presented superior short-term outcomes when compared to both another minimally invasive technique, the direct anterior approach, and conventional approaches [53, 54]. The direct anterior approach is another very popular technique for THA in recent years because of its proven advantages reported in the literature [55–58]. Nevertheless, Ramadanov et al. reported that the SuperPath reduced the operation time, incision length, intraoperative blood loss, and early pain intensity compared to the direct anterior approach [54]. Several criticisms were made towards different MIS regarding the correct placement of the acetabular cup, due to not always clear surgical visualization of the acetabulum [15, 18, 45, 59]. Recent literature supports the use and effectiveness of MIS. Good results of IA and AA with the SuperPath and direct anterior approach were obtained, despite a slight tendency towards a flat IA (IA through the SuperPath with a range of 37.1° to 43.8°) [54]. Our results demonstrate satisfactory acetabular cup positioning through the SuperPath technique, with an average IA of 42,7° ± 8,2 (an average of 82% cases over 4 years in the Lewinnek safe zone), and an average AA of 17,6° ± 3,2 (an average of 77% cases over 4 years in the Lewinnek safe zone) in a total of 756 radiographic cases. We inspected the radiographic results of acetabular cup placement reported in the literature by authors comparing SuperPath with conventional surgical approaches (Table 6).

**Table 6:** Comparison of SuperPath with conventional surgical approaches reported in the recent literature

	Surgical approaches	Number of patients	IA (mean ± SD) (°)	AA (mean ± SD) (°)
**Xie et al. [23]**	SuperPath	46	43,6 ± 6,8	17,4 ± 1,6
	Conventional posterior	46	44,5 ± 6,5	18,5 ± 1,8
**Ouyang et al. [48]**	SuperPath	12	37,08 ± 6,53	21,92 ± 5,78
	Posterolateral	12	39, 67 ± 6,95	21,75 ± 4,48
**Meng et al. [59]**	SuperPath	4	38, 75 ± 8,21	15 ± 1,82
	Posterolateral	4	44,5 ± 3,64	14,25 ± 2,06
**Meng et al. [46]**	SuperPath	20	36,94 ± 6,37	13,94 ± 4,73
	Posterolateral	20	42,66 ± 3,58	15,11 ± 4,06
**Tottas et al. [60]**	SuperPath	25	51,2 ± 4,8	20,5 ± 9,8
	Hardinge	23	43,77 ± 4,4	25 ± 7,9

In the systematic review and meta-analysis by Ramadanov et al., 80 patients operated by SuperPath technique and 80 patients operated by conventional approaches, collected from 4 randomized controlled trials, were evaluated. The results of this study showed no difference regarding the acetabular cup placement [22]. On the other hand, Tottas et al. reported, in a group of 48 patients, IA values with SuperPath statistically higher than mean IA values obtained with the Hardinge approach (51,2° ± 4,8 vs 43,7° ± 4,4, respectively); while statistically similar values were found in the two groups regarding AA (20,5° ± 9,8 in the SuperPath group vs 25,0° ± 7,9 in the Hardinge approach group) [60]. Evaluating the above IA and AA values of these authors, our results show substantially similarities. We obtained satisfactory values of IA and AA in a large case series (756 THAs) regarding the safe zone proposed by Lewinnek, with a low number of hip dislocations (0,3%), moreover in the first year of the study. In addition, analyzing the averages of inclination angle across years, it was noted that there was no statistically significant improvement with increased surgical experience with the SuperPath technique. This finding can be interpreted in two ways: the learning curve for obtaining a good cup placement does not require few years to obtain satisfactory radiographic results; conversely, with the SuperPath technique, a statistically better result cannot be achieved in a 4-year period, even with increased surgical experience. This confuted the second hypothesis of our study.

Our study has a few limitations, such as the retrospective nature of the results, lack of randomization between orthopaedic surgeons and operated patients, lack of a control group with conventional surgical approach, additional clinical information of patients (e.g., BMI, postoperative lower limb dysmetria). In addition, the measurement of AA with conventional radiographs is questionable since the use of computed tomography provides a more accurate measurement and is not affected by the pelvic position [34, 53]. Finally, there is no correlation between radiographic and several clinical results, but this was not the aim of our study, and the numerous benefits of SuperPath in clinical terms were already reported in the literature [21, 23, 24, 34, 54, 61].

## Conclusions

The SuperPath technique allows to obtain in experienced surgeons of conventional postero- lateral approach, who underwent fellowship of this muscle sparing technique, satisfactory radiographic results of acetabular cup placement in THA, with a low rate of hip dislocation. It does not take many years to achieve such results. These outcomes are statistically consistent over a 4-year period. Further studies with more cases and longer follow-up are needed, to further investigate the validity of the SuperPath technique in a more comprehensive clinical-radiographic fashion.

## Data Availability

The datasets used and/or analysed during the current study are not publicly available because they are deemed essential for use in further studies in the future, but they are available from the corresponding author on reasonable request.

## Abbreviations

SuperPath: Supercapsular percutaneously assisted total hip
THA: Total hip arthroplasty
IA: Inclination angle
AA: Acetabular anteversion
LLD: Leg length discrepancy
FO: Femoral offset
AO: Acetabular offset
SD: Standard deviation
MIS: Surgical minimally invasive
PACS: Picture Archiving and Communication System
